# The ten-year evolutionary trajectory of a highly recurrent paediatric high grade neuroepithelial tumour with *MN1:BEND2* fusion

**DOI:** 10.1038/s41598-018-19389-9

**Published:** 2018-01-18

**Authors:** Anna Burford, Alan Mackay, Sergey Popov, Maria Vinci, Diana Carvalho, Matthew Clarke, Elisa Izquierdo, Aimee Avery, Thomas S. Jacques, Wendy J. Ingram, Andrew S. Moore, Kieran Frawley, Timothy E. Hassall, Thomas Robertson, Chris Jones

**Affiliations:** 1 Centre for Evolution and Cancer, Institute of Cancer Research, London, UK; 2 Division of Molecular Pathology, Institute of Cancer Research, London, UK; 3 Division of Cancer Therapeutics, Institute of Cancer Research, London, UK; 4 Department of Pathology, Cardiff University School of Medicine, Cardiff, UK; 5 Bambino Gesù Children’s Hospital, Rome, Italy; 6 Department of Histopathology, Great Ormond Street Hospital for Children NHS Foundation Trust, London, UK; 7 Developmental Biology and Cancer Programme, UCL GOS Institute of Child Health, London, UK; 8 UQ Child Health Research Centre, The University of Queensland, Brisbane, Australia; 9 The University of Queensland Diamantina Institute, Translational Research Institute, Brisbane, Australia; 10 Oncology Services Group, Children’s Health Queensland Hospital and Health Service, Brisbane, Australia; 11 Medical Imaging and Nuclear Medicine, Children’s Health Queensland Hospital and Health Service, Brisbane, Australia; 12 Pathology Queensland, Royal Brisbane and Women’s Hospital, and School of Medicine, University of Queensland, Brisbane, Australia

## Abstract

Astroblastomas are rare brain tumours which predominate in children and young adults, and have a controversial claim as a distinct entity, with no established WHO grade. Reports suggest a better outcome than high grade gliomas, though they frequently recur. Recently, they have been described to overlap with a newly-discovered group of tumours described as’high grade neuroepithelial tumour with MN1 alteration’ (CNS HGNET-MN1), defined by global methylation patterns and strongly associated with gene fusions targeting *MN1*. We have studied a unique case of astroblastoma arising in a 6 year-old girl, with multiple recurrences over a period of 10 years, with the pathognomonic *MN1:BEND2* fusion. Exome sequencing allowed for a phylogenetic reconstruction of tumour evolution, which when integrated with clinical, pathological and radiological data provide for a detailed understanding of disease progression, with initial treatment driving tumour dissemination along four distinct trajectories. Infiltration of distant sites was associated with a later genome doubling, whilst there was evidence of convergent evolution of different lesions acquiring distinct alterations targeting NF-κB. These data represent an unusual opportunity to understand the evolutionary history of a highly recurrent childhood brain tumour, and provide novel therapeutic targets for astroblastoma/CNS HGNET-MN1.

## Introduction

Astroblastomas are rare glial tumours of unknown origin^1^. These lesions are characterised by the presence of the perivascular pseudorosettes composed of tumour cells with a prominent process extending to a central blood vessel and perivascular hyalinization. In the previous (2007) WHO classification no formal grade was recommended^2^, although well-differentiated and malignant variants have been recognized^3^, possibly with clinical consequences^4^. Astroblastomas are positive for GFAP (glial fibrillary acidic protein), S100 and vimentin by immunohistochemistry and frequently display focal expression of EMA (epithelial membrane antigen)^4^. They predominantly occur supratentorially with differential diagnoses including high grade glioma, ependymoma, AT/RT (atypical teratoid/rhabdoid tumour) and CNS-PNET (primitive neuroectodermal tumour)^1^. These lesions most commonly present in children and young adults, with a clear female predominance^5^ and may have a better clinical outcome than glioblastoma^6^. Astroblastomas have mostly been described in case reports, and their status as a distinct clinicopathological entity has been controversial.

In the last few years, large-scale molecular profiling studies of multiple childhood brain tumour types has revealed novel insights into the taxonomy of cancers occurring within the CNS^7^. Specifically, CNS-PNETs have recently been resolved by biological subclassification using 450k methylation arrays into either multiple well-known brain tumour pathologies or one of four new molecular entities^8^. These novel entities differ in their anatomical location, age at incidence and histological features, and are marked by distinct pathognomonic structural variants, and are described as CNS neuroblastoma with *FOXR2* activation (CNS NB-*FOXR2*), CNS Ewing sarcoma family tumour with *CIC* alteration (CNS EFT-*CIC*), CNS high-grade neuroepithelial tumour with *BCOR* alteration (CNS HGNET-*BCOR*), and CNS high-grade neuroepithelial tumour with *MN1* alteration (CNS HGNET-*MN1*). This latter group was specifically associated with gene fusions involving *MN1* (22q12.3), usually with *BEND2* (Xp22.13), but also the alternative partner *CXXC5* (5q31.2), and contained 16/23 cases diagnosed as astroblastoma by the originating centres.

One of the samples in the study described above included a case of astroblastoma diagnosed in a 6 year-old girl who suffered ten recurrences of her tumour over a ten-year period before finally succumbing to her disease. Multiple recurrences have been reported previously in isolated cases of astroblastomas with specific high grade histological features^9^, although the rarity of the disease and lack of available tissue has precluded biological analyses of this phenomenon. We were fortunate to have access to tumour tissue from all 11 surgical interventions from this patient for exome sequencing, which has allowed us the unique opportunity to study the clonal evolution of this novel molecular entity over a long period in the childhood setting, and in response to different treatment strategies.

## Materials and Methods

### Case and samples

All patient material was collected after informed consent and subject to the Children’s Health Queensland Human Research Ethics Committee local research ethics committee approval, and all procedures performed in accordance with relevant guidelines and regulations. Formalin-fixed paraffin-embedded (FFPE) tumours samples were available for all 11 resections (termed **A**–**I**) with frozen tumour available for 4 of these (**E**, **F**, **H** and **I**). Detailed histopathological assessment was carried out on haematoxylin and eosin stained sections. Pre-gadolinium sagittal and axial T1, axial T2 FLAIR and diffusion weighted scans of the whole brain were performed with post gadolinium coronal and axial T1 weighted scans also acquired.

### Immunohistochemistry

Standard 4 µm formalin fixed paraffin embedded histologic sections of tumour were stained with the antibodies, EMA clone E29 at 1:100 (Dako Agilent, Santa Clara, CA, USA), GFAP polyclonal at 1:1000 (Dako Agilent, Santa Clara, CA, USA), and S100 polyclonal at 1:2000, (Dako Agilent, Santa Clara, CA, USA), using a Leica Bond III (Leica Biosystems) automated immunohistochemical stainer. Staining for NF-κB p65 D14E12 (Cell Signalling, The Netherlands) was undertaken on a Leica Bond Max automated immunohistochemical stainer with protocol F, at an antibody concentration of 1:500, after heat induced epitope retrieval at pH 6 for 20 minutes.

### DNA extraction and sequencing

At least 400ng DNA was sent for exome sequencing at the Tumour Profiling Unit, ICR, London, UK using the 50 Mb Agilent SureSelect platform (Agilent, Santa Clara, CA, USA), and paired-end-sequenced on an Illumina HiSeq. 2000 (Illumina, San Diego, CA, USA) with a 100 bp read length. Reads were aligned to the hg19 build of the human genome using bwa v 0.7.5a (bio-bwa.sourceforge.net), and PCR duplicates removed with PicardTools 1.5 (pcard.sourceforge.net). Somatic single nucleotide variants were called using the Genome Analysis Tool Kit v3.3-0 based upon current Best Practices using local re-alignment around indels, downsampling and base recalibration with variants called by the Unified Genotyper (broadinstitute.org/gatk). Variants were annotated using the Ensembl Variant Effect Predictor v71 (ensembl.org/info/docs/variation/vep) incorporating SIFT (sift.jcvi.org) and PolyPhen (genetics.bwh.harvard.edu/pph2) predictions, COSMIC v64 (sanger.ac.uk/genetics/CGP/cosmic) and dbSNP build 137 (ncbi.nlm.nih.gov/sites/SNP) annotations. Copy number was obtained by calculating log_2_ ratios of tumour/normal coverage binned into exons of known genes, smoothed using circular binary segmentation (bioconductor.org) and processed using in-house scripts. We also determined the somatic allele-specific copy number profiles using read depth from whole genome/ exome sequencing as above analysed by ASCAT^10^, which also provided for an estimate of the non-neoplastic cell contamination of the sample as well as the overall ploidy of the tumour. Loss of heterozygosity (LOH) was also calculated using ASCAT based upon a minor allele frequency < 0.2. Allele-specific copy number, LOH and tumour cell purity were then used to calculate the cancer cell fraction (CCF) which estimates the percentage of tumour cells carrying each mutation^11^, and truncated to 100% where experimental variability in sequence reads produced a value greater than this figure. Intratumoral heterogeneity and the number and frequency of sub-populations within individual tumour samples were calculated with the EXPANDS algorithm using evolutionary biology principles including the Shannon and Simpson indices, and allowing for the concept that subclones may share a subset of variants that may be nested within each other^12^. All data are deposited in the European Genome-phenome Archive (ebi.ac.uk/ega) under accession number EGAS00001002432.

### RNA extraction and sequencing

RNA was extracted from frozen tumour tissue following the RNeasy Plus Mini Kit protocol (Qiagen), quantified on a 2100 Bioanalyzer (Agilent Technologies), and sequenced on an Illumina GA-II genome analyser as 100 bp paired end reads. RNA sequences were aligned to hg19 and organised into de-novo spliced alignments using bowtie2 and TopHat2 (https://ccb.jhu.edu/software/tophat). Raw read counts and fragments per kilobase per million reads mapped (FPKPM) were calculated for all known Ensembl genes in assembly v74 using bedtools (bedtools.readthedocs.org) and Cufflinks (cole-trapnell-lab.github.io/cufflinks/). Putative fusions were identified using Chimerascan 0.4.5 (code.google.com/archive/p/chimerascan/). Published RNAseq data was taken from Mackay *et al*.^13^, whilst published Affymetrix gene expression array data was taken from Sturm *et al*.^8^.

### MN1:BEND2 RT-PCR

Total RNA was extracted frozen samples using the Qiagen RNeasy Plus Mini Kit. cDNA was synthesized using the SuperScript II system (Life Technologies) according to manufacturer’s protocol. Four PCR primers (Life Technologies) were designed using Primer3, ± 200 bp from the breakpoints to generate amplicons spanning the breakpoint-junction-sequences of the predicted fusion. The following primers were used: *MN1* Fwd1 - CAGCAGTTCAGCATCTCCGA, *BEND2* Rev1 - TTGTACGGCCAGCAAATGGA, *MN1* Fwd2- CATTGACCTGGACTCGCTGA, *BEND2* Rev2 - TGACGCTGTTTTCCGTGTTG. cDNA from KNS42 was used as control. PCR was carried out with Platinum Taq DNA Polymerase High Fidelity (Life Technologies) in a 25 μl volume using a 2-Step-Touchdown programme: (94 °C 30 sec, 68 °C 45 sec, 72 °C 1 min, 18 cycles; 94 °C 30 sec, 50 °C 45 sec, 72 °C 1 min, 30 cycles), and cleaned up with the IllustraTM ExoStarTM 1-STEP kit (VWR). Sanger DNA sequencing of the PCR products was performed by DNA Sequencing & Services (MRCPPU, College of Life Sciences, University of Dundee, Scotland, dnaseq.co.uk) using Applied Biosystems Big-Dye Ver 3.1 chemistry on an Applied Biosystems model 3730 automated capillary DNA sequencer.

### MN1:BEND2 FISH

BAC clones were purchased from BACPAC Resources Center (Children’s Hospital Oakland Research Institute, Oakland, CA, USA) and FISH-mapped onto metaphase slides to ensure specificity. BACs used were: *MN1* (FITC / DIG) - RP11-145O03, RP11-166A15; *BEND2* (Spectrum Orange / biotin) - RP11-768A24, RP11-46N14. BAC DNA was amplified from 10ng starting material using the IllustraTM GenomiPhi V2 DNA amplification kit (GE Healthcare) according to the manufacturer’s instructions. Probes were labelled with either biotin or DIG using the BioPrime DNA Labelling System (Life Technologies). Tissue slides were stripped of paraffin using xylene, pre-treated with 0.2 M HCL and 8% sodium thiocyanate for 30 minutes or Spot-light pre-treatment buffer (Life Technologies) at 80 °C for 1hr, digested in 0.025% pepsin (Sigma) in 0.01 M HCL at 37 °C for 20–70 minutes or twice in Digest-All pepsin solution (Life Technologies) for 21 minutes before adding a prepared probe mix with (75%) hybridization buffer under a coverslip and denatured for 5 minutes at 80 °C on a heat block. They were then incubated overnight in a humidified chamber at 37 °C, mounted in Vectashield with DAPI (Vector Laboratories, Peterborough, UK), and captured on the Metafer Scanning and Imaging platform (MetaSystems, Altlussheim, Germany) at x60 using filters for DAPI, Cy3 and FITC.

## Results

### Initial presentation

**Figure 1:**
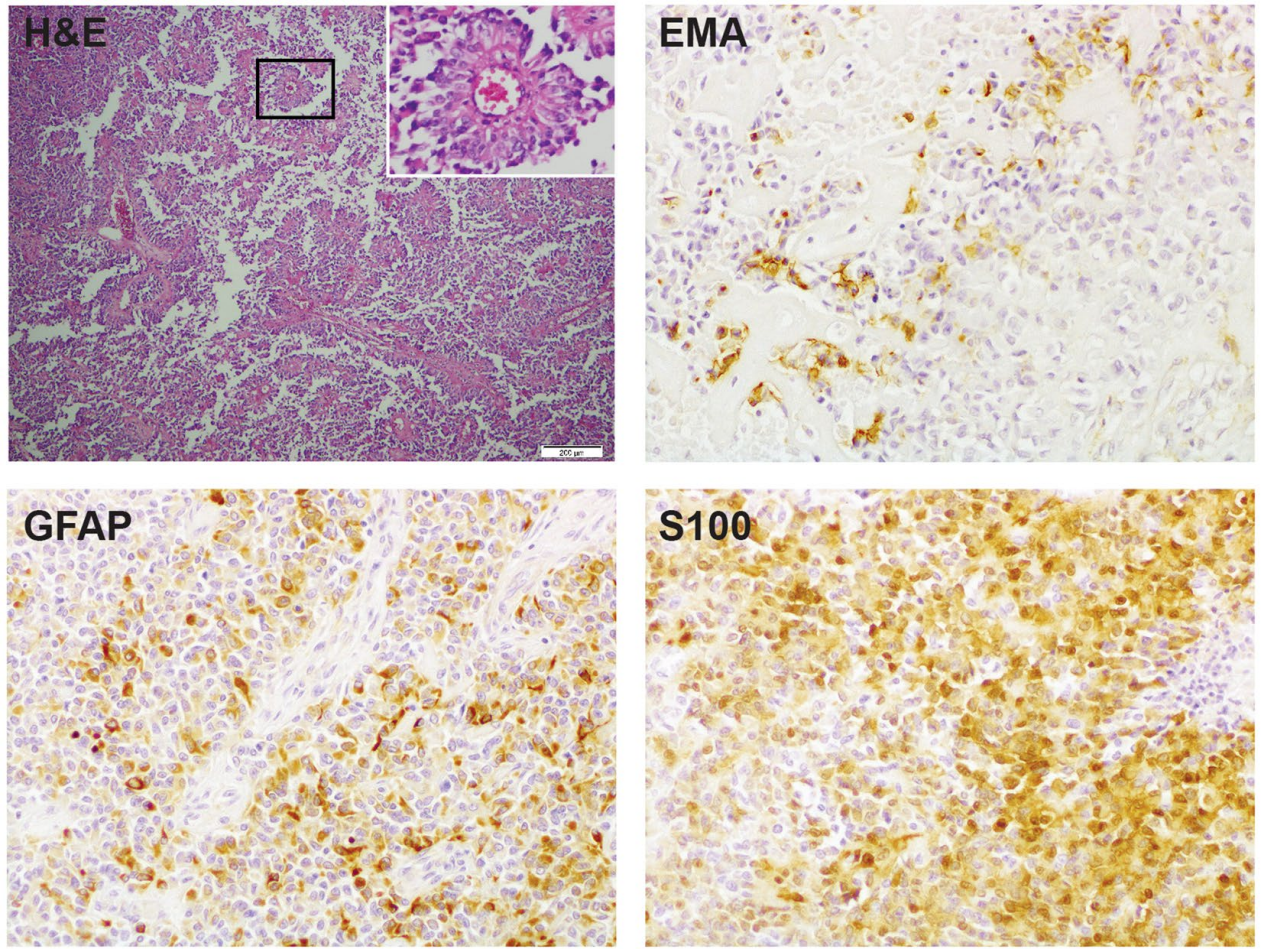
Histopathological features at presentation. Sample. (**A)** with haematoxyllin and eosin (H&E) staining, there are areas of multiple perivascular pseudorosettes (inset) as well as areas of necrosis and vascular hyalinisation. Cells are heterogenously positive for EMA, GFAP and S100 by immunohistochemistry. Original magnification x100 (inset, x400).

A six-year old girl presented with recurring headaches, and upon magnetic resonance imaging (MRI) a cystic and solid mass within the superior aspect of the left parietal lobe anterior to the central sulcus was observed (Specimen **A**). After surgical resection, histological examination revealed an unusual tumour with solid, solid papillary and papillary histological growth patterns. Tumour cells were radially orientated around often hyalinised vascular walls, forming perivascular pseudo-rosettes. There was focal necrosis and numerous mitoses (up to 28 per 10 high power fields). The tumour cells were positive for vimentin and exhibited patchy positive staining for GFAP and S-100, as well as focal positivity for EMA (Fig. 1). Sections were negative for SMA, MSA, desmin, myogenin, myoglobin, neurofilament protein and cytokeratin 34 Beta E12 (not shown). Conventional cytogenetics identified loss of an X chromosome and partial deletion of 22q. Differential diagnoses included high grade glioma, anaplastic ependymoma and rhabdoid papillary meningioma, but the pathologic features were thought to be most consistent with an astroblastoma with malignant features. No adjuvant chemotherapy was administered after gross total resection.

### Recurrences

**Figure 2:**
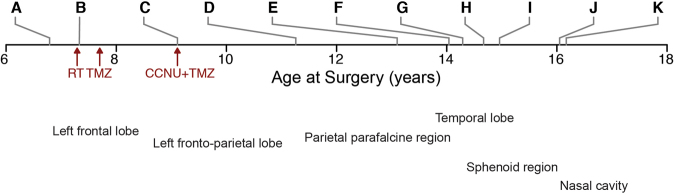
Clinical timeline. Surgical samples are lablled. (**A**–**K)**, and annoated by age at resection/biopsy and anatomical location. Points of treatment initiation are indicated in red. RT, radiotherapy; TMZ, temozolomide; CCNU, lomustine.

**Figure 3:**
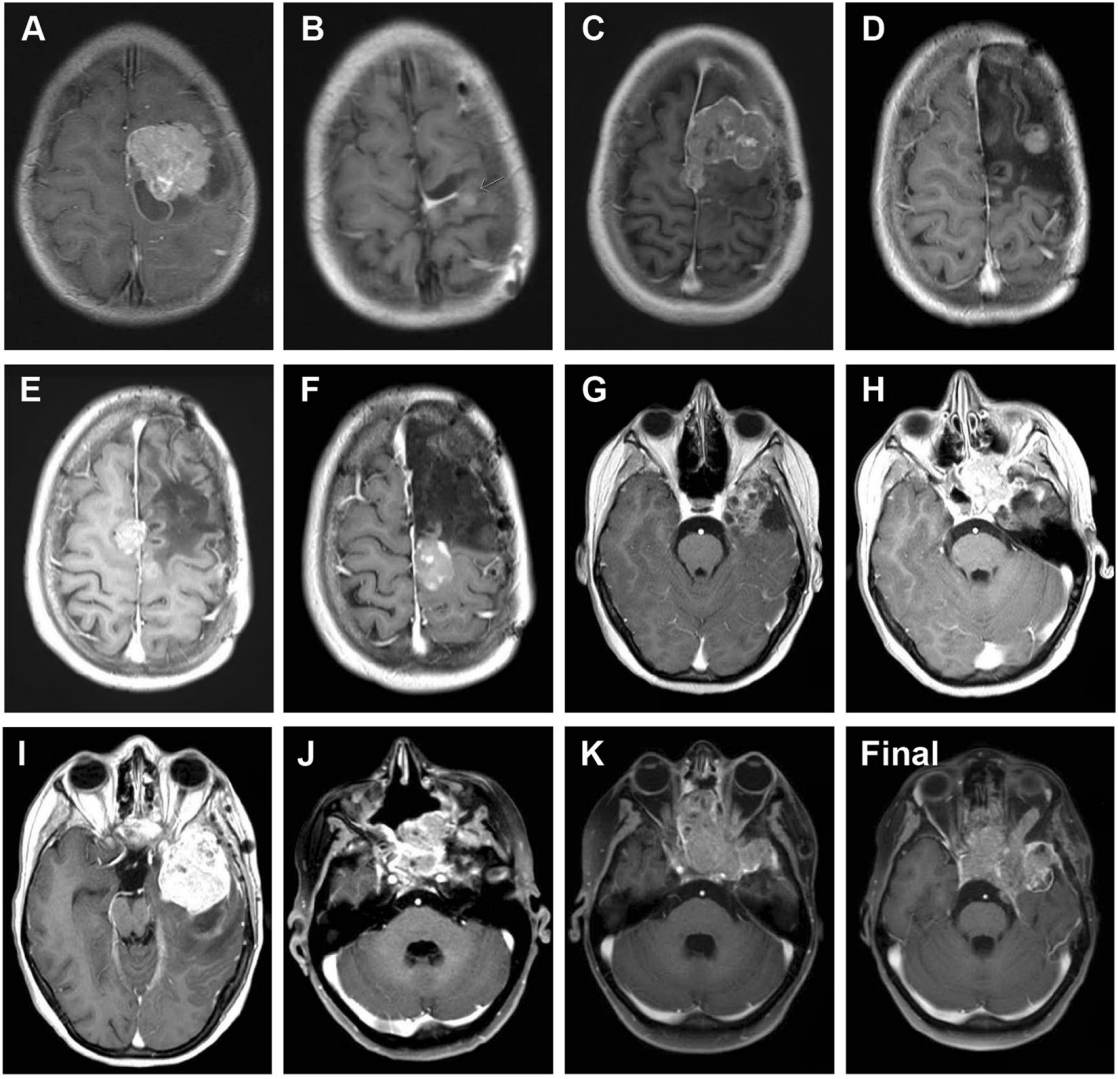
Post-contrast, T1 weighted axial MRI images of all recurrences. (**A**–**K**) representative images prior to 1^st^ to 11^th^ surgical resections respectively. (**L**), representative image of final tumour progression. (**A**) age 6.79, underwent surgical resection of left frontal tumour. (**B**) age 7.33, left frontal craniotomy, partial resection, treated with radiotherapy and temozolomide. (**C**) age 9.11, left frontal subtotal resection, treated with CCNU and temozolomide. (**D**) age 11.26, biopsy. (**E**) age 13.10, resection. **(F)** age 14.04, resection. (**G**) age 14.29, right temporal craniotomy, gross total resection. (**H**) age 14.67, sphenoid tumour, endoscopic endonasal sphenoidectomy. (**I**) age 14.96, left temporal resection. (**J**) age 16.05, nasal biopsy. (**K**) age 16.17, nasal cavity resection. The patient died aged 16.42 years (Final).

**Figure 4:**
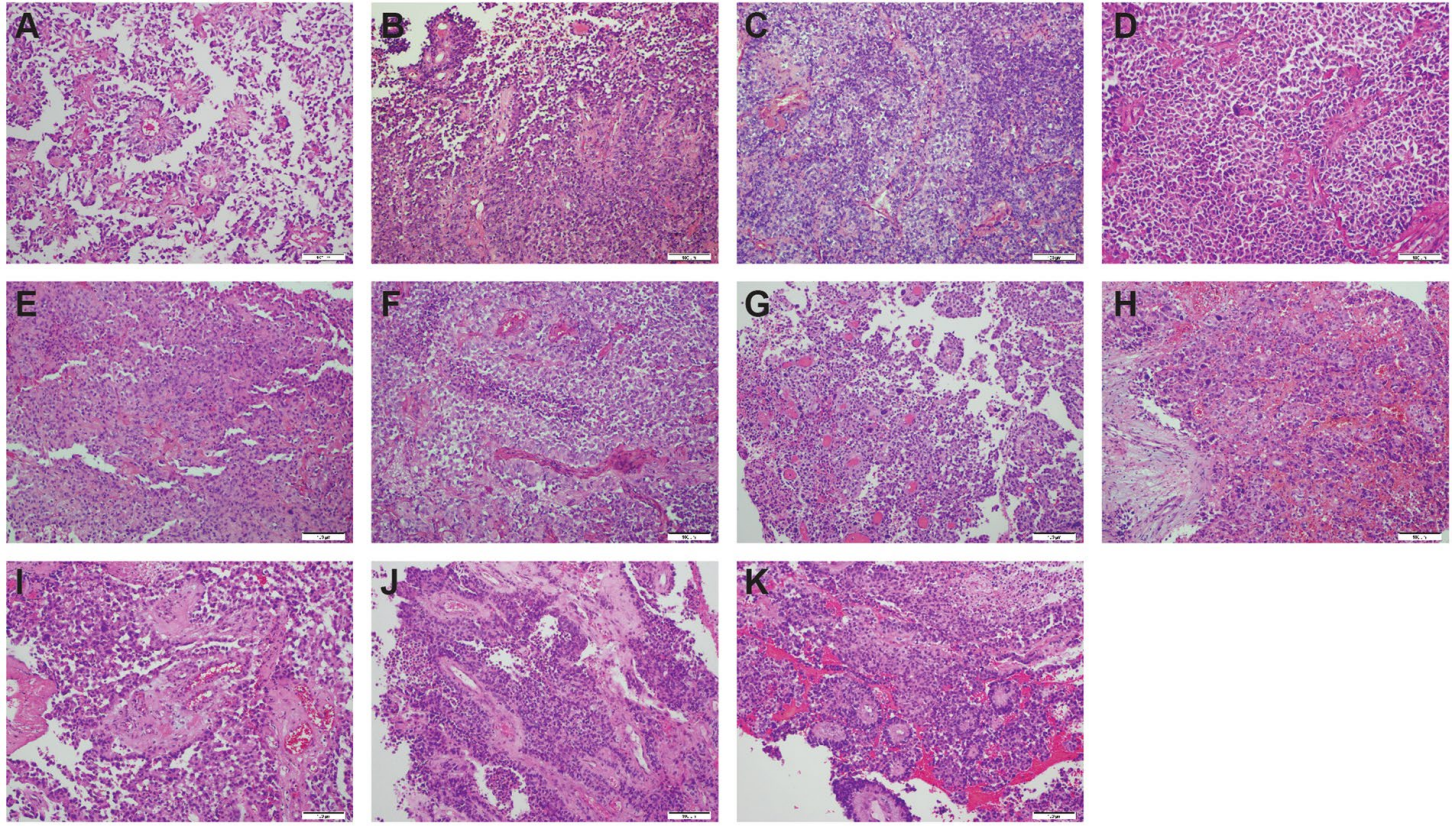
Histopathological features of all recurrences. (**A**) Initial presentation – age 6.79 years (as in Fig. 1). (**B**) Similar morphology with high focal mitotic activity, fewer perivascular pseudorosettes and sharply demarcated borders. Evidence of calcification. (**C**) More cellular tumour with only single ribbon-like structures and almost no pseudorosettes seen. (**D**) Similar morphology to **C**, with hyalinised vessels. (**E**) Fewer mitotic figures present, and cells seen with conspicuous nucleoli. (**F**) Similar to (**B**), but with no calcification. Increased perivascular ribbons and necrotic foci. (**G**) Distinct lesion with prominent cellular pleomorphism and large hyperchromatic nuclei. Many cells have an epithelioid appearance. There are papillary structures and numerous pseudorosettes. (**H**) Tumour is infiltrating the sphenoid bone and sinus and has numerous invasive, pleomorphic cells with hyperchromatic and large nuclei. (**I**) Well vascularised pleomorphic tumour with several pseudorosettes. (**J**) Tumour was mostly necrotic tumour but viable tissue. showed pseudorosettes around hyalinised vessels. (**K**) Tumour was mostly necrotic. but preserved tumour shows numerous pseudorosettes, mostly around hyalinised vessels with thick walls. Original magnification x200.

Over the course of the next 10 years, the patient underwent a further 10 invasive procedures to remove a highly recurrent tumour (Fig. 2). She was treated with chemoradiotherapy with temozolomide after the second surgery at age 7.33 years (**B**), and combined CCNU/temozolomide after a third surgery at age 9.11 years (**C**). Subsequent to this, later recurrences were removed without further radio- or chemotherapy. A representative T1 weighted axial MRI series for all 11 lesions and a final progression image is provided (Fig. 3). The anatomical locations ranged from the left frontal/fronto-parietal lobes (**A-E**), parietal parafalcine region (**F**), temporal lobe (**G, I**), sphenoid region (**H**) and nasal cavity (**J, K**). The histology for all samples were consistent with that of recurrent anaplastic astroblastoma, with a varying degree of atypical mitoses, pseudorosettes, hyalinised vessels and necrosis (Fig. 4). An exception is the seventh surgery (**G**) which was the most pleomorphic of the samples, and appeared upon radiological review to be an entirely separate lesion away from the site of the primary tumour and considered to represent a local metastasis. The patient died aged 16.42 years.

### Identification of MN1:BEND2 fusion

**Figure 5:**
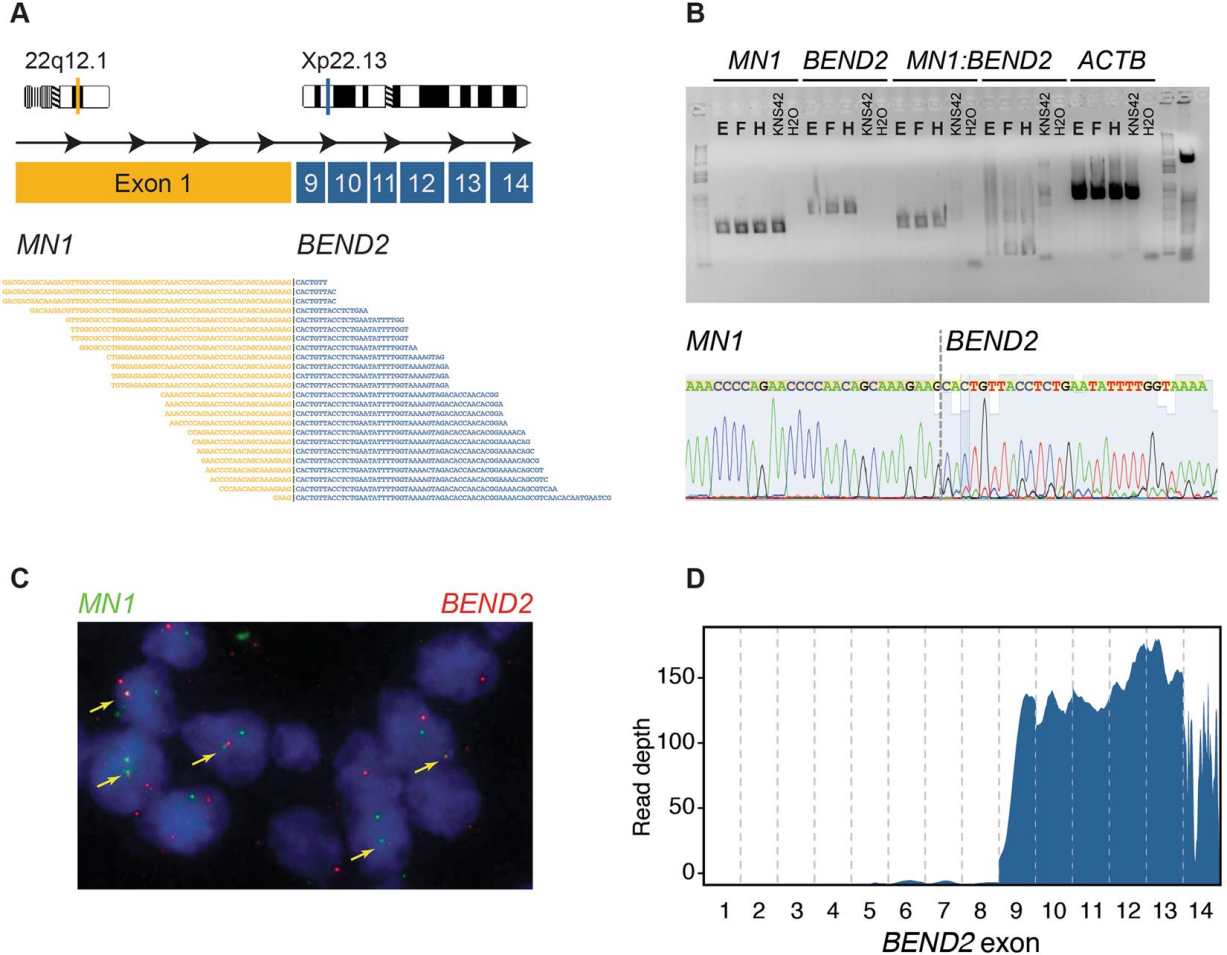
Identification of MN1:BEND2 fusion. (**A**) Cartoon of fusion between exon 1 of *MN1* (22q12.1, orange) and exons 9–14 of *BEND2* (Xp22.13, blue), with spanning reads from RNAseq from sample (**F**) underneath. (**B**) Top: RT-PCR using primers designed against *MN1*, *BEND2*, and spanning the breakpoint, in samples (**E**), (**F**) and (**H**). KNS42 paediatric glioblastoma cells were used as a fusion-negative control. Beta actin (*ACTB*) was used as a positive RT-PCR control. Bottom: Sanger sequencing of the fusion band in sample. (**E**) Showing a sequence spanning the breakpoint. (**C**) FISH using probes directed against *MN1* (green) and *BEND2* (red), detecting fusion signals throughout sample (**H**). (**D**) RNAseq coverage of *BEND2*, with high levels of expression of exons 9–14 in sample (**E**).

FFPE material was available for all 11 specimens, with three tumours having sufficient frozen sample (**F**, **H**, **I**), providing material for RNA sequencing. Analysis for fusion transcripts in these latter samples showed a consistent apparent inter-chromosomal translocation between *MN1* (meningioma disrupted in balanced translocation 1, 22q12.1) and *BEND2* (BEN domain containing 2, Xp22.13) (Fig. 5A). This structural variation involves a deletion at chromosome 22q and breakpoints in intron 1 of *MN1* and intron 8 of *BEND2*, rearrangement, and results in an expressed transcript comprising exon 1 of *MN1* and exons 9 though 14 of *BEND2*. This was validated in the frozen samples by RT-PCR (Fig. 5B), and the FFPE specimens by custom FISH probes (Fig. 5C) (Supplementary Figure S1). The consequence of this fusion is increased expression of *BEND2*, as seen by exon-level sequence coverage by RNA sequencing (Fig. 5D).

### Tumour evolution

**Figure 6:**
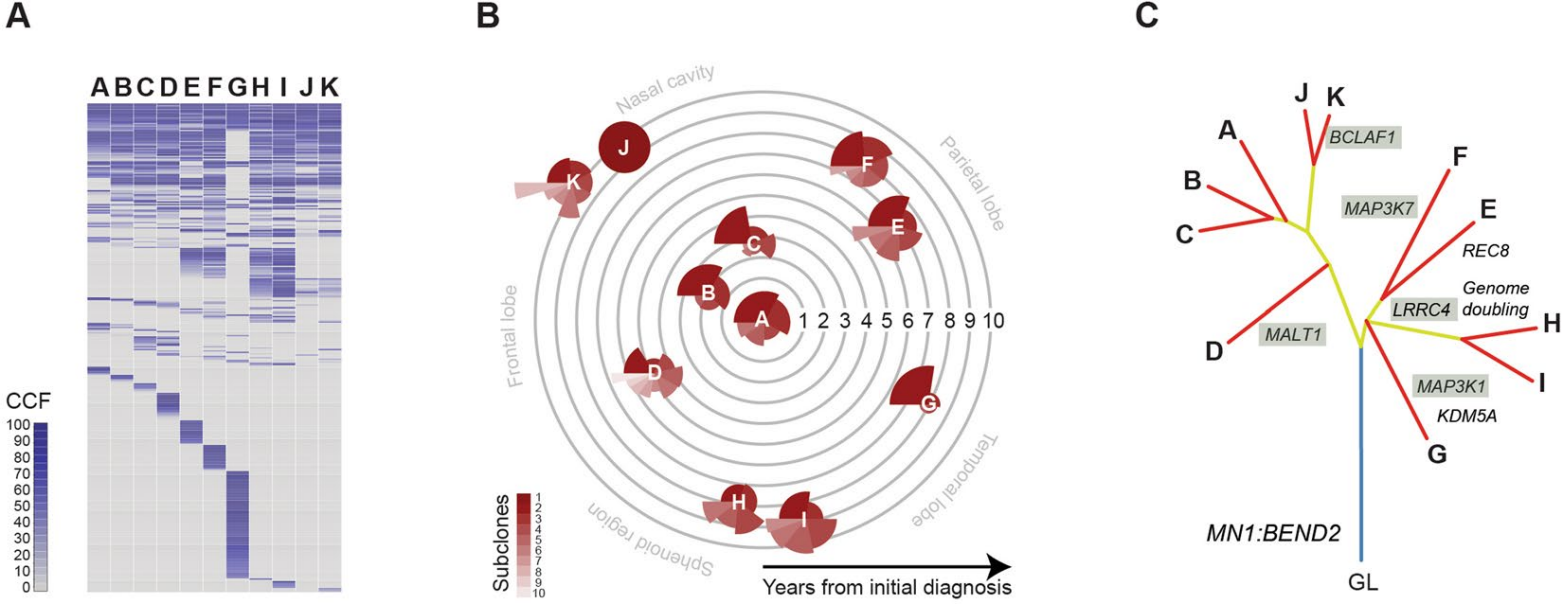
Intratumoral heterogeneity and phylogentic reconstruction. (**A**) Contingency plots for cancer cell fractions (CCF) of somatic mutations identified in specimens (**A**–**K**). Key as provided in the figure. (**B**) Coxcomb plots for number of subclones in each specimen, with the area of each scaled according to the number of variants predicted to be contained within, as calculated by EXPANDS. They are placed along concentric circles according to the sampling time after initial diagnosis, and in distinct anatomical locations. (**C**) Phylogenetic tree of tumour specimens built using maximum parsimony from exome sequencing data. Blue line represents the ‘trunk’, yellow lines the shared major and minor ‘branches’, red lines the private ‘leaves’. Selected alterations are labelled, those targeting the NF-κB pathway are shaded.

Exome sequencing was carried out on all surgical specimens (Supplementary Figure S2). A total of 110 somatic single nucleotide variants (SNVs) and InDels were found in any one or more of the samples (4–36 per specimen, median = 13). The highest number were found in resection **G** (n = 36). Calculating the cancer cell fractions (CCFs) for the somatic variants allowed for a temperospatial reconstruction of the tumour phylogeny based upon common, shared and private events (Fig. 6A). Aside from the *MN1:BEND2* fusion, only a single common mutation was observed in all specimens – a small deletion (p.1211delQ) in *GIGYF2* (GRB10 interacting GYF protein 2).

The interpretation of multi-sample sequencing data can be heavily influenced by the extent of intratumoral heterogeneity present across the tumour(s). To assess this, we investigated the subclonal architecture of each specimen using EXPANDS (Fig. 6B). The number of major subclones ranged from 4–10, with the exception of specimen **J**, which appeared to be largely clonal, and notably distinct from the multi-clonal specimen **K**. Strikingly different compositions from the initial presentation were also observed in the spatially distinct specimens **D** (a considerably more complex subclonal architecture compared to **A, B** and **C)** and **G** (dominated by a predominant clone) (Fig. 6B).

Upon phylogenetic reconstruction, the samples were split in two main branches (Fig. 6C). The first included the primary presentation within the frontal lobe (**A**) and the closely-related local recurrences **B** and **C**. These were found to have few chromosomal alterations and 8–10 somatic SNVs/InDels. Specimen **D**, resected from the fronto-parietal region, was distinguished by several additional alterations including missense variants in the paracaspase *MALT1* and acquisition of gain of chromosome 13q. Notably, the other samples in this branch were from the latest surgeries acquired from the nasal cavity (**J** and **K**), which shared the relatively stable copy number genome and few mutations, though **K** had acquired several additional variants including the BCL2-associated transcription factor *BCLAF1*.

The remaining samples were found on a second major branch (Fig. 6C). Sample **G** formed a branch separate from the other resections, reflecting its distinct radiopathological features, location within the temporal lobe, and with few chromosomal changes but a large number of private mutations including missense mutations in the serine/threonine kinase *MAP3K1* and lysine demethylase *KDM5A*. The other four samples were associated with the acquisition of a large number of DNA copy number changes, particularly whole chromosomal arm gains, as well as mutation in *LRRC4*. Two specimens resected from the parafalcine region (**E**) and the vertex superior to the frontal lobe (**F**) formed their own sub-branch, the former acquiring a truncating mutation in the meiotic recombination protein *REC8*, the latter a missense variant in *MAP3K7*. Finally, a further sub-branch was found to contain the remaining tumours **H** and **I**, resected from the sphenoid region and temporal lobe, respectively.

**Figure 7:**
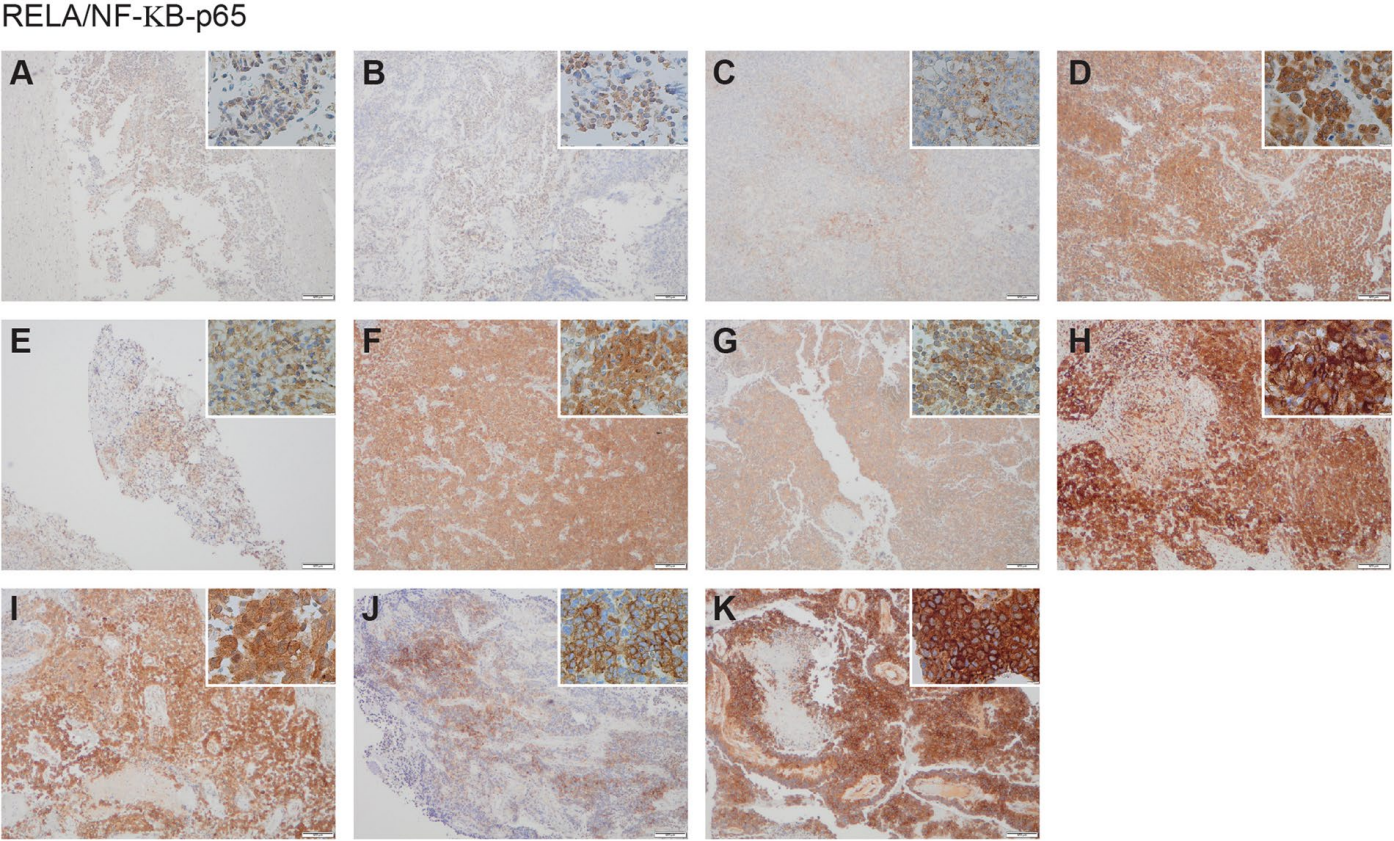
NF-κB pathway activation. Strong cytoplasmic and nuclear expression of RELA/NF-κB-p65 by immunohistochemistry is seen in all later specimens (**D**–**K**), and only weakly observed in (**A**–**C**). Main image, original magnification x200; inset x400.

The concordance of these distinct sub-branches having apparently independently acquired mutations in genes associated with NF-κB signalling (*MALT1, BCLAF1, MAP3K1, LRRC4* and *MAP3K7*) prompted us to investigate pathway activation by immunohistochemistry for RELA-p65 (Fig. 7). Whilst only very weak staining was observed in specimens **A**, **B** and **C** (absent these mutations), all other tumours showed strong nuclear-cytoplasmic accumulation of staining indicative of NF-κB signalling. This was associated with the increased expression of the target gene *IL8* in specimens for which RNAseq data was available, compared to a series of hemispheric high grade glioma (HGG) samples (Supplementary Figure S3A). In an independent series of brain tumour samples, *IL8* gene expression was found to be at equivalent levels to *C11orf95-RELA* fusion-positive ependymoma, and significantly elevated compared to AT/RT, HGG and medulloblastoma.

**Figure 8:**
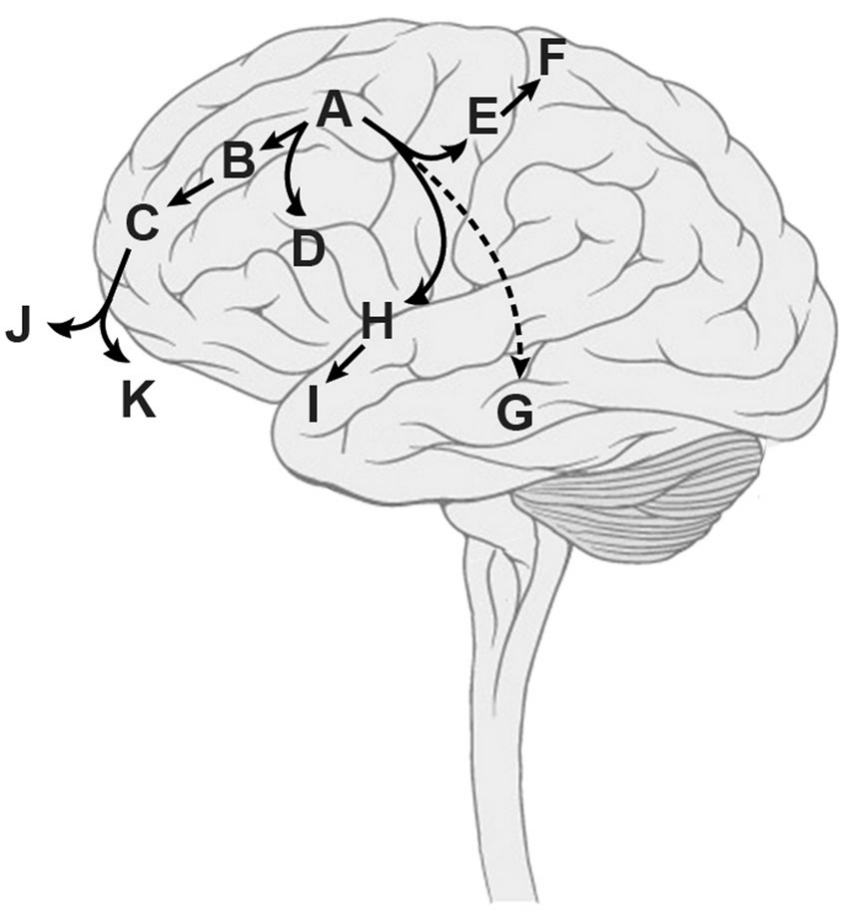
Integrated clinical and molecular inference of tumour evolution. Taking all available data, we proposed an evolutionary history along four distinct trajectories over 10 years. Brain diagram modified from Patrick J. Lynch, medical illustrator; C. Carl Jaffe, MD, cardiologist, under Creative Commons Attribution 2.5 License 2006. (https://upload.wikimedia.org/wikipedia/commons/4/44/Brain_human_lateral_view.svg).

Interpreting the molecular and clinical data together, we propose an evolutionary history whereby after acquisition of the primary driving *MN1:BEND2* fusion (**A**), there was an early spread of tumour cells from the original site after initial chemoradiotherapy and surgery, with divergence along several pathways (Fig. 8). The first involved little genomic instability, with local recurrence in the frontal lobes (**B**, **C**) and an evolutionarily independent spread to parietal regions (**D**), followed >5 years later by eventual migration such that the latest surgical interventions were in the nasal cavity (**J**, **K**). These latter two samples had remarkably different subclonal compositions, suggestive of distinct temporal origins. In parallel were three metastatic routes involving distinct anatomical regions. The first appears to be an early escape to the temporal lobe (**G**), with few overlapping somatic alterations with the primary tumour, and predominantly clonal. The latter two seem to occur after the acquisition of major chromosomal changes reminiscent of genome doubling, and diverged along either superior (**E**, **F**) or inferior (**H**, **I**) paths over the course of 18 months.

## Discussion

Since it’s first description by Bailey and Cushing in 1924^14^, there has been controversy as to whether the histopathological features which are suggestive of the term astroblastoma mark a distinct entity^2^. There have historically been few biological studies of astroblastoma, which has made resolution of its pathogenesis difficult. The largest study, from Brat and co-workers in 2000, carried out DNA copy number profiling by comparative genomic hybridization (CGH) on 7 cases, and reported frequent gains of chromosome 19 and 20q, as well as losses of 9q, 10 and X^15^. Prior to this, karyotyping of a case from a 15 year old girl revealed monosomies of chromosomes 10, 21 and 22^16^. More recently, a lack of *IDH1*^17^ and *TP53*^18^ mutations in astroblastomas has been noted but there have been no characteristic genetic features reported giving positive clues as to their likely origin^19^.

Recent integrated molecular analysis of another childhood brain tumour with controversial claims to being a disinct entity, CNS-PNET, have helped to clarify these issues, with the identification of a novel molecular entity, CNS HGNET-MN1, in which 16/41 cases were initially diagnosed as astroblastoma. This group of tumours had the oldest age at diagnosis, a largely supratentorial location, a striking female:male predominance, and the best overall survival of all novel entities described^8^. Histologically they had features overlapping with that descibing astroblastoma (*e.g*. dense perivascular hyalinization) but not all cases could be so rationalised. They expressed GFAP but lacked neuronal antigen expression.

The most striking feature of the newly described CNS HGNET-MN1 group were the pathognomonic fusions of *MN1* (and *BEND2*, or in one case, *CXXC5*)^8^. The highly recurrent case described in the present paper harboured the most common fusion, *MN1:BEND2*, resulting in expresson of a transcript containing the large exon 1 of *MN1* with exons 9–14 of the longest transcript of *BEND2*. This was seen in all 11 surgical resection samples in our case, indicating an early, clonal, drive event in the pathogenesis of this tumour. Althought the function of BEND2 is unknown, a consequence of the mutation is the high expression levels of the two BEN-domains encoded by the gene, thought to play a role in chromatin organisation^20^ and neural transcriptional regulation^21^. As other fusion partners of *MN1* have been reported^8^, disruption of this gene may instead be the pathogenic event. *MN1* is frequently targeted by a balanced t(4,22) translocation certain subtypes of meningioma, and acts as a transcriptional co-regulator of retinoic acid-mediated gene expression along with p300^22,23^.

Although generally considered to harbour a good prognosis, extensively recurrent cases have been previously reported^9^. Exome sequencing of the multiple recurrences which occurred in the patient presented here afforded us the opportunity to infer a molecular evolution of the disease which aligned with the clinical presentation. This involved an early divergence from the original site after initial treatment, disseminating via four distinct anatomical routes to multiple locations within the brain. At a relatively advanced stage of presentation, this involved a doubling of the tumour genome not seen in the initial local recurrences. Interestingly, the final biopsies obtained via the nasal cavity were found not to contain these chromosomal changes, and were rather two further independent infiltrative extensions of the original local recurrence-derived branch.

In all but the very earliest of the surgical samples we analysed, in addition to the truncal *MN1:BEND2* fusion we identified variants in genes associated with NF-κB signalling, and concurrent pathway activation as evidenced by immunohistochemistry for RELA-p65. These included *MAP3K1*, a downstream target of Bcr-Abl reported to mediate the anti-apoptotic effects of NF-κB^24^; *MAP3K7*, which phosphorylates the IKK complex, leading to eventual release of inhibition and activation of NF-κB^25^; *MALT1*, which has both scaffolding functions and proteolytic activity essential for NF-κB activation^26^; *BCLAF1*, a direct target of p65 and c-Rel, and essential for C/EBPβ upregulation^27^; and *LRRC4*, a candidate tumour suppressor gene down-regulated in gliomas which appears to play a role in cellular proliferation by mediating MAPK/PI3K and NF-κB signalling^28^. NF-κB has long been thought to play a role in glioma phenotypes^29^, and more recently emerged as a critical regulator in ependymoma^30^. The observation that these mutations occurred in the later specimens suggests a possible role in progression of CNS HGNET-MN1 tumours, and also provides a plausible therapeutic target.

## Electronic supplementary material

Supplementary Information
