# Supplementary material for: The ten-year evolutionary trajectory of a highly recurrent paediatric high grade neuroepithelial tumour with MN1:BEND2 fusion
Source: Sci Rep. 2018 Jan 18;8:1032. doi: 10.1038/s41598-018-19389-9 (PMC5773598; doi:10.1038/s41598-018-19389-9)

SUPPLEMENTARY INFORMATION

for

The ten-year evolutionary trajectory of a highly recurrent paediatric high grade neuroepithelial tumour with *MN1:BEND2* fusion

Anna Burford, Alan Mackay, Sergey Popov , Maria Vinci, Diana Carvalho, Matthew Clarke, Elisa Izquierdo, Aimee Avery, Thomas S Jacques, Wendy J Ingram, Andrew S Moore, Kieran Frawley, Timothy E Hassall, Thomas Robertson and Chris Jones

Supplementary Figure S1 – *FISH for MN1:BEND2 fusion in all recurrences*. FISH using probes directed against *MN1* (green) and *BEND2* (red), highlighting fusion signals in samples **A-K**.

MN1:BEND2 fusion probes

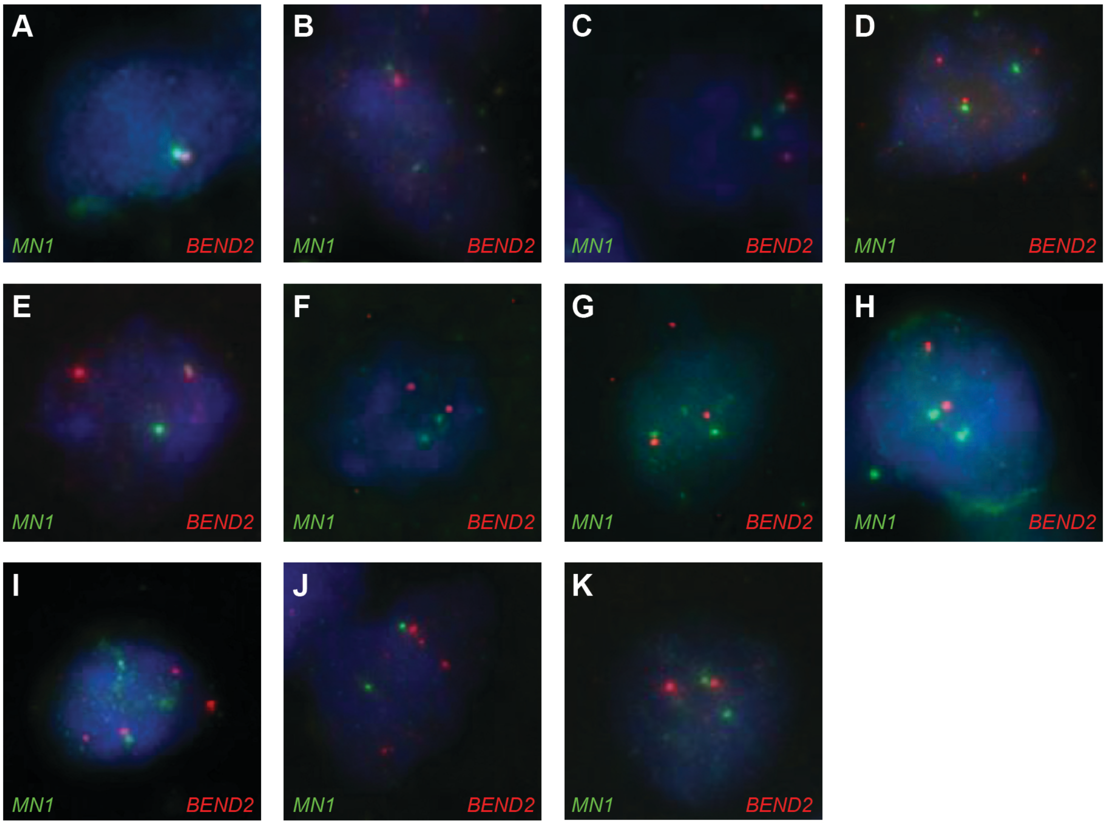

Supplementary Figure S2 – *Exome sequencing of all recurrences*. For each sample **A-K**, a CIRCOS plot is given highlighting somatic SNVs and InDels (outer ring), DNA copy number changes (red=gain, blue=loss) and loss of heterozygosity (yellow) on the inner rings, and inter-chromosomal translocations inside the circle (blue).

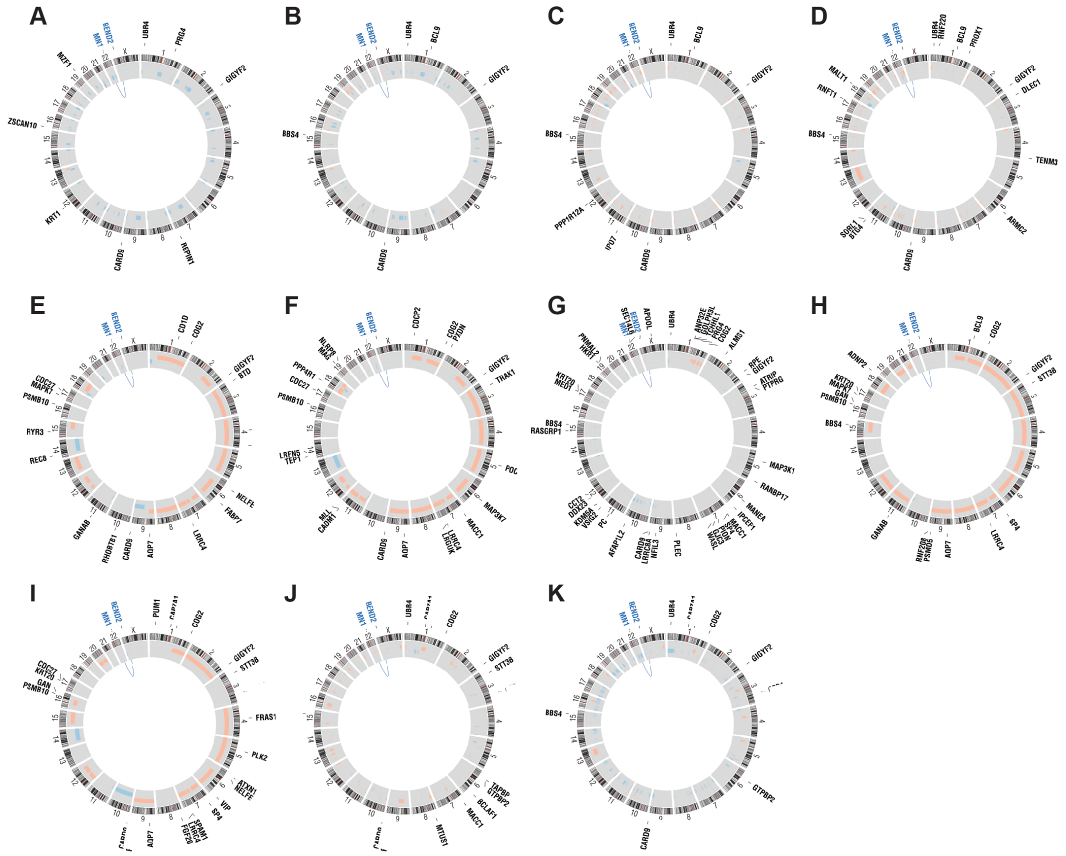

Supplementary Figure S3 – *Expression of IL8 in HGNET-MN1.* (A) Boxplots of RNA sequencing data from the current reference case where available (**E**, **F**, **H** and **I**) compared with a published series of hemispheric high grade glioma (HGG)[13]. (B) Boxplots of Affymetrix U133Plus2.0 expression array data of a series of atypical teratoid / rhabdoid tumours (AT/RT), hemispheric high grade glioma (HGG), medulloblastoma (MG), RELA-fusion positive ependymoma (EPN-RELA) and HGNET-MN1[8]. ***p<0.0001, t-test

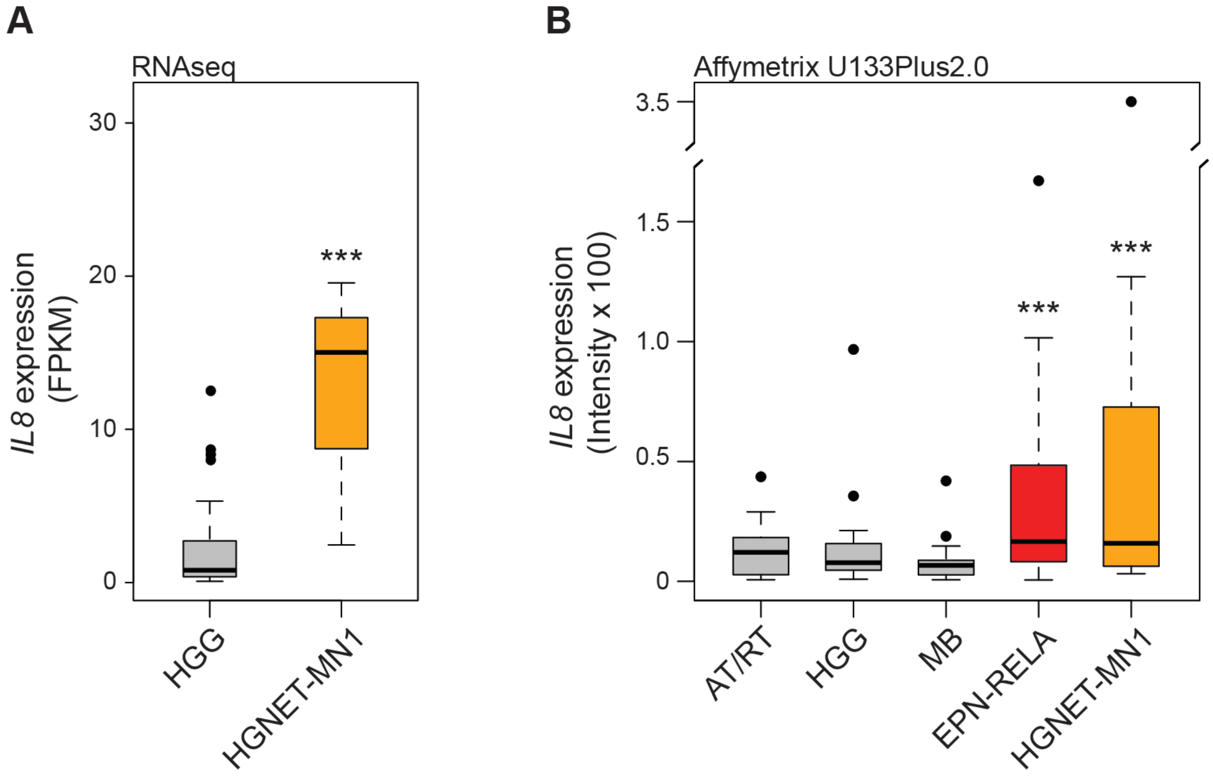

Supplement: Supplementary file 1 — Supplementary Information [file 41598_2018_19389_MOESM1_ESM.pdf]
